# Supplementary material for: A multi-modal transformer for predicting global minimum adsorption energy
Source: Nat Commun. 2025 Apr 4;16:3232. doi: 10.1038/s41467-025-58499-7 (PMC11971357; doi:10.1038/s41467-025-58499-7)
Supplement: Supplementary file 1 — Supplementary information [file 41467_2025_58499_MOESM1_ESM.pdf]

Supplementary information for

A multi-modal transformer for predicting global minimum adsorption energy

Junwu Chen[1,2†], Xu Huang[1,3†], Cheng Hua[4], Yulian He[3,5*] and Philippe Schwaller[1,2*]

[1]*Laboratory of Artificial Chemical Intelligence (LIAC), Institute of Chemical Sciences and Engineering, Ecole Polytechnique Fédérale de Lausanne (EPFL), Lausanne, Switzerland*

[2]*National Centre of Competence in Research (NCCR) Catalysis, Ecole Polytechnique Fédérale de Lausanne (EPFL), Lausanne, Switzerland*

[3]*Department of Chemistry and Chemical Engineering, Shanghai Jiao Tong University, Shanghai, China*

[4]*Antai College of Economics and Management, Shanghai Jiao Tong University, Shanghai, China*

[5]*University of Michigan–Shanghai Jiao Tong University Joint Institute (UM-SJTU JI), Shanghai, China*

[†]*These authors contributed equally to this work.*

[*]*Corresponding authors. Email: yulian.he@sjtu.edu.cn; philippe.schwaller@epfl.ch*

Supplementary Notes

N1 Dataset description . . . S5

N2 Dimensionality reduction of GMAE datasets . . . S6

N3 Adsorption site identification . . . S7

N4 Error curve at various uncertainty cutoffs . . . S8

N5 Calibration curve and miscalibration area . . . S9

N6 Uncertainty recalibration . . . S10

N7 Computational cost comparison . . . S11

N8 Hyperparameters for AdsMT models . . . S12

N9 Similarity between OC20-LMAE and GMAE datasets . . . S13

N10 Identify different types of adsorption sites from cross-attention scores . . . S14

N11 Sensitivity of the AdsMT model to surface geometric fluctuations . . . S15

Supplementary Figures

S1 The periodic table outlining elements included in the Alloy-GMAE dataset. . . . S16

S2 The periodic table outlining elements included in the FG-GMAE dataset. . . . S17

S3 The periodic table outlining elements included in the OCD-GMAE dataset. . . . S18

S4 The periodic table outlining elements included in the OC20-LMAE dataset. . . . S19

S5 A brief illustration of the Alloy-GMAE dataset construction process. . . . S20

S6 A brief illustration of the FG-GMAE dataset construction process. . . . S21

S7 A brief illustration of the OCD-GMAE dataset construction process. . . . S22

S8 Eight examples comparing top views of cross-attention score-colored surfaces (left) and global minimum adsorption structures optimized by density functional theory (right), where the cross-attention scores are computed by the trained AdsMT model adopting GemNet-OC graph encoder. The color bar depicts the value of the cross-attention score of the surface atoms. . . . S23

S9 Eight examples comparing top views of cross-attention score-colored surfaces (left) and global minimum adsorption structures optimized by density functional theory (right), where the cross-attention scores are computed by the trained AdsMT model adopting AdsGT graph encoder. The color bar depicts the value of the cross-attention score of the surface atoms. . . . S24

S10 Eight examples comparing top views of cross-attention score-colored surfaces (left) and global minimum adsorption structures optimized by density functional theory (right), where the cross-attention scores are computed by the trained AdsMT model adopting eSCN graph encoder. The color bar depicts the value of the cross-attention score of the surface atoms. S25
S11 Eight examples comparing top views of cross-attention score-colored surfaces (left) and global minimum adsorption structures optimized by density functional theory (right), where the cross-attention scores are computed by the trained AdsMT model adopting ET graph encoder. The color bar depicts the value of the cross-attention score of the surface atoms. S26
S12 Uncertainty estimation of AdsMT models with different graph encoders on the GMAE datasets: (a) Cumulative MAE at different cutoffs of uncertainty percentiles, and corresponding (b) Spearman correlation coefficients between the estimated uncertainty and cumulative MAE. S27
S13 Calibration curves (a) of AdsMT models with different graph encoders after recalibration on the GMAE datasets, and corresponding (b) miscalibration area. S28
S14 Examples of identifying different adsorption site types based on the extended method and cross-attention scores. S29

Supplementary Tables

S1 Overview of three new GMAE datasets. S30
S2 Adsorbate information for Alloy-GMAE dataset S30
S3 Adsorbate information for FG-GMAE dataset S31
S4 Adsorbate information for OCD-GMAE dataset S32
S5 List of adsorbate descriptors used in the AdsMT model S33
S6 Test MAE results (eV) of AdsMT framework with different graph encoders on the three GMAE datasets without transfer learning. The best results for each GMAE dataset are marked in bold. S36
S7 Test success rate results (%) of AdsMT framework with different graph encoders on the three GMAE datasets without transfer learning. The best results for each GMAE dataset are marked in bold. S37
S8 Performance comparison of MAE (unit: eV) and success rate (SR, unit: %) for AdsMT models adopting different graph encoders w/o and with transfer learning (TL) on the Alloy-GMAE dataset. S38

S9 Performance comparison of MAE (unit: eV) and success rate (SR, unit: %) for AdsMT models adopting different graph encoders w/o and with transfer learning (TL) on the FG-GMAE dataset. S38
S10 Performance comparison of MAE (unit: eV) and success rate (SR, unit: %) for AdsMT models adopting different graph encoders w/o and with transfer learning (TL) on the OCD-GMAE dataset. S39
S11 Hyperparameters for the AdsMT model with AdsGT graph encoder on the Alloy-GMAE dataset. S40
S12 Hyperparameters for the AdsMT model with AdsGT graph encoder on the FG-GMAE dataset. S41
S13 Hyperparameters for the AdsMT model with AdsGT graph encoder on the OCD-GMAE dataset. S42
S14 Mean absolute energy differences (eV) of GMAE predictions between original and noise-perturbed surface structures using the same trained models. S43
S15 Test MAE (eV) results of AdsMT models using different dataset splitting methods on the Alloy-GMAE dataset S44
S16 Test success rate (SR, %) results of AdsMT models using different dataset splitting methods on the Alloy-GMAE dataset. S44
S17 Test MAE (eV) results of AdsMT models using different dataset splitting methods on the FG-GMAE dataset. S45
S18 Test success rate (SR, %) results of AdsMT models using different dataset splitting methods on the FG-GMAE dataset. S45
S19 Comparison of the success rate (%) and computational speed (comb/min) of AdsMT and pretrained Uni-Mol+ on the OCD-GMAE dataset. S46

Supplementary Notes

N1 Dataset description

Three new GMAE datasets, named Alloy-GMAE, FG-GMAE and OCD-GMAE, were built from Catalysis Hub [1], 'functional groups' (FG)-dataset [2], and OC20-Dense [3] datasets, respectively. Each of the source datasets enumerated all adsorption sites on surfaces and performed DFT calculations on various possible adsorption configurations. The data cleaning was conducted to remove abnormal adsorption structures and take the lowest adsorption energy of all conformations as the GMAE target for each surface/adsorbate combination.

For the Alloy-GMAE dataset, the catalyst surfaces were generated from bimetallic alloys and pure metals. The bimetallic alloys were chosen by combining 37 selected metals (outlined in the periodic table in Fig. S1) to form alloys in the $L1_2$ and $L1_0$ Strukturbericht designation, which corresponds to face-centered cubic crystal structures with A_3B and AB stoichiometries, respectively. The 37 pure metals in the $A1$ (FCC) structure were included in addition to the bimetallic alloys resulting from all possible combinations, such that stoichiometric A:B ratios of 0 %, 25 %, 50 %, 75 %, and 100 % are sampled. The catalyst surfaces were modeled by cleaving three-layer slabs with a (111) termination for $A1$ and $L1_2$ and a (101) termination for $L1_0$. As shown in Table S2, atomic adsorbates of H, C, N, O, and S were included, as well as hydrogenated species CH, NH, CH_2, CH_3, SH, OH and H_2O.

FG-GMAE includes 202 organic molecules and 14 transition metals (Ag, Au, Cd, Co, Cu, Fe, Ir, Ni, Os, Pd, Pt, Rh, Ru, and Zn) with the lowest surface-energy facets. As shown in Fig. S2, metals include eight face-centered cubic (fcc), one body-centered cubic, and five hexagonal closed packed, and (111), (110), and (0001) surfaces. As depicted in Table S3, the included adsorbates span the most common functional groups in organic chemistry containing N, O, and S heteroatoms: (1) non-cyclic hydrocarbons; (2) O-functionalized (alcohols, ketones, aldehydes, ethers, carboxylic acids, and carbonates); (3) N-functionalized (amines, imines, and amidines); (4) S-functionalized (thiols, thioaldehydes, and thioketones); (5) N- and O-functionalized combinations (amides, oximes and carbamate esters); and (6) aromatic molecules with up to two rings, also containing heteroatoms.

OCD-GMAE consists of 973 combinations spanning 967 inorganic surfaces (intermetallics, ionic compounds, etc.), coupled with 74 adsorbates (O/H, C_1, C_2, N-based). The surfaces involve 54 elements (Fig. S3) including alkali/alkaline earth metals, transition/post-transition metals, metalloids, and reactive nonmetals. The adsorbates contain 74 types and 4 elements (Table S4). The catalyst surfaces and adsorbates were sampled from the Open Catalyst 2020 (OC20) dataset [4].

N2 Dimensionality reduction of GMAE datasets

Dimensionality reduction was carried out via the uniform manifold approximation and projection (UMAP) algorithm [5, 6] as implemented in umap-learn v0.5.5. UMAP constructs a weighted graph of three GMAE datasets in the high-dimensional space and then projects this graph to a two-dimensional space. Each node of the graph represents a surface/adsorbate combination, and each combination is depicted by the smooth overlap of atomic positions (SOAP) descriptors [7, 8] of surfaces concatenated with RD-Kit descriptors [9] of adsorbates. The number of neighbors was set to 50, and the minimum distance between points was set to 0.4. All other parameters were set to the default values (for reproducibility, a random seed of 16 was used).

SOAP is a descriptor that encodes regions of atomic geometries by using a local expansion of a Gaussian smeared atomic density with orthonormal functions based on spherical harmonics and radial basis functions. In this work, average SOAP descriptors of catalyst surface were generated by DScribe v2.1.0 package [10, 11]. One way of turning a local SOAP descriptor matrix into a global descriptor vector is simply by taking the average over all sites. The inner averaging mode in DScribe was used, which is taken over the sites before summing up the magnetic quantum number based on:

$$p_{nn'l}^{Z_1,Z_2} \sim \sum_m \left(\frac{1}{n} \sum_i c_{nlm}^{i,Z_1} \right) \left(\frac{1}{n} \sum_i c_{n'lm}^{i,Z_2} \right), \tag{S1}$$

$$c_{nlm}^{Z} = \iiint_{\mathcal{R}^3} \mathrm{d}V g_n(r) Y_{lm}(\theta, \phi) \rho^Z(\mathbf{r}), \tag{S2}$$

where n and n' are indices for the different radial basis functions up to n_{max}, l is the angular degree of the spherical harmonics up to l_{max}, Z_1 and Z_2 are atomic species, and $\rho^Z(\mathbf{r})$ is the Gaussian smoothed atomic density for atoms. In general, the inner averaging will preserve the configurational information better [10, 11]. Additionally, the cutoff for the local region is set to 4.0 Å, the number of radial basis functions is set to 8, the maximum degree of spherical harmonics is set to 6, and the feature compression method is set to mu2.

N3 Adsorption site identification

In this study, we explored the application of cross-attention scores to speculate the most energetically favorable adsorption sites on catalyst surfaces. The AdsMT architecture utilizes separated information of catalyst surfaces and adsorbates to predict GMAE, and no information related to adsorption configurations or sites was provided for model training. As introduced in the methods section (Equation 10-14), the cross-attention scores of head n are calculated based on:

$$\text{score}_n = \text{softmax}\left(\frac{\boldsymbol{q}_n \mathbf{K}_n^T}{\sqrt{2k}}\right). \tag{S3}$$

Then, the average cross-attention scores from all heads is computed according to:

$$\hat{\text{score}} = \frac{1}{N}\sum_{n=1}^{N} \text{score}_n, \tag{S4}$$

where N is the total head number in a cross-attention layer. The $\hat{\text{score}}$ calculated by the last cross-attention layer of the trained AdsMT model is employed to infer the optimal adsorption site for each surface/adsorbate combination. The surface atom(s) with the highest average cross-attention score is hypothesized as the most favorable adsorption site. The atom embeddings are updated based on neighboring atoms in the graph encoder, so equivalent atoms on the catalyst surface have nearly the same cross-attention scores.

The ground truths of the most energetically favorable adsorption sites are obtained from the DFT-optimized adsorption structures at GMAE. Each true adsorption site is represented by a set Ω of surface atoms, where each atom binds with the adsorbate. For a certain surface atom i, if there is an adsorbate atom j such that the distance d_{ij} between them is less than the threshold c_{ij}, the surface atom i is considered to be bound to the adsorbate based on:

$$d_{ij} \leq r_i + r_j + \delta, \tag{S5}$$

where r_i and r_j are the covalent radii of the atom i and j, and δ is a tolerance parameter fixed at 1.2 Å.

The accuracy of adsorption site identification is calculated based on the surface atoms with the highest average cross-attention scores and the true adsorption site Ω from the DFT-optimized adsorption structures at GMAE. A correct speculation is defined as the presence of the surface atom with the highest average cross-attention score, or its equivalent atom, within the set Ω (true adsorption site). If the set Ω is empty, this surface/adsorbate combination is disregarded. Although this definition is not strict, it can effectively evaluate the ability of cross-attention scores to reason about the most favorable adsorption sites. Moreover, we also calculated the baseline accuracy of adsorption site speculation based on random atom selection rather than the atom with the highest cross-attention score. The baseline accuracies on the Alloy-GMAE and OCD-GMAE are 0.083 and 0.049, respectively.

N4 Error curve at various uncertainty cutoffs

One method for assessing uncertainty estimation involves examining its alignment with prediction errors. A robust uncertainty estimation should yield low errors, particularly within the subset of high-confidence predictions. We followed an evaluation scheme [12] to test whether the estimated uncertainties correlate with prediction errors. We sorted predictions $\hat{y}_i$ in decreasing order represented by index $\{r_i\}$, such that $\hat{y}_{r_i}$ has the i-th highest estimated uncertainty (i.e., $\hat{y}_{r_1}$ is the most uncertain prediction while $\hat{y}_{r_n}$ is the most confident prediction, where n is the total number of test samples). For every value i, we computed the cumulative mean absolute error (MAE) for the test samples $\left\{\hat{y}_{r_j} : j \geq i\right\}$, i.e., $\mathrm{MAE}_i = \frac{1}{n-i}\sum_{j\geq i}^{n} |\hat{y}_{r_i} - y_{r_i}|$, where y_{r_i} is the ground-truth value corresponding to the predicted value $\hat{y}_{r_i}$. This metric evaluates the model's prediction accuracy at various confidence cutoffs. For example, setting $i = 0.5n$ gives the MAE at the 50 % confidence cutoff. We plotted the cumulative MAE as a function of different confidence cutoffs.

N5 Calibration curve and miscalibration area

We also consider calibration as a crucial and more stringent criterion for uncertainty estimation. Calibration assesses whether the magnitude of uncertainty estimates corresponds to statistically meaningful confidence intervals. While the ensemble-based approach to uncertainty quantification in deep neural networks has proven effective, it has been observed that these uncertainty estimates can sometimes lack calibration, resulting in either over-confidence or under-confidence [13, 14]. For example, if we interpret the prediction and uncertainty as the mean and the variance of a Gaussian distribution $\mathcal{N}\left(\mu\left(\boldsymbol{x}_i\right), \sigma\left(\boldsymbol{x}_i\right)^2\right)$ and consider its 95 % confidence interval, an over- (or under-) confident model would produce an interval containing the true data points less (or more) than 95 % of the time, and a well-calibrated model predicts an interval where true data points would fall within this interval 95 % of the time.

To evaluate the calibration of uncertainty estimates, we followed a widely used procedure used in previous studies [12, 15, 16]. We interpreted the predicted GMAE $\mu\left(\boldsymbol{x}_i\right)$ and its uncertainty $\sigma\left(\boldsymbol{x}_i\right)$ of a data point x_i as the mean and standard deviation of a Gaussian distribution $\mathcal{N}\left(\mu\left(\boldsymbol{x}_i\right), \sigma\left(\boldsymbol{x}_i\right)^2\right)$. Given a confidence interval of level e, we computed the e % confidence interval boundaries of data point x_i using the inverse CDF function F_i^{-1} of Gaussian distribution defined by $\mu\left(\boldsymbol{x}_i\right)$ and $\sigma\left(\boldsymbol{x}_i\right)$, i.e., $L_i^e = F_i^{-1}(0.5 - e/2)$ and $R_i^e = F_i^{-1}(0.5 + e/2)$. For a calibrated model, we would expect that the fraction of ground truth data points falling in that interval is e. To find the empirical or observed fraction, we count the fraction of ground-truth data points falling in the interval, that is $\hat{f}_e = \left|\{y_i \mid L_i^e \leq y_i \leq R_i^e\}\right| / N_D$, where N_D is the number of data points. Plotting $\hat{f}_e$ against various e for $0 \leq e \leq 1$ gives a curve called a calibration curve. The calibration curve of a perfectly calibrated predictive model is the diagonal line. Therefore, to quantify the degree of uncertainty calibration, we computed the area between the model's calibration curve and the parity line, which is called the miscalibration area. We used Uncertainty Toolbox v0.1.1 package [17] to calculate the calibration curves and miscalibration areas.

N6 Uncertainty recalibration

There are two widely recognized definitions of regression calibration in the literature: confidence-interval-based calibration [14] and error-based calibration [18]. Under confidence-based calibration, a model is said to be well-calibrated if e % of its predictions fall in the e % predicted confidence interval ($(0 \leq e \leq 1)$) [14], whereas error-based calibration defines a well-calibrated model as one for which the uncertainty estimate of a prediction, in expectation, equals the prediction errors [18]. Several approaches have been proposed to recalibrate regression models [12, 14, 18]. The general idea is to learn a post hoc transformation function, which receives the model's predicted uncertainties as input and outputs the transformed uncertainty estimates that would be better calibrated. We use a simple effective scaling approach [17, 18] to recalibrate the uncertainty estimates. Specifically, we transform the model's output $(\mu(x_i), \sigma(x_i))$ to $(\mu(x_i), r\sigma(x_i))$, where r is the scaling factor to be learned. Note that the model's prediction of GMAE $\mu(x_i)$ does not change. To learn the scaling factor r, we introduce an optimization problem in which the objective is to minimize the miscalibration area. The recalibration is a post hoc process, meaning the model's predicted uncertainties are fixed and only r is optimized. As indicated previously [14], the recalibration is performed on a held-out validation set that has not been used for model training. We use Brent's method [19] to solve this single-variable optimization. All uncertainty recalibrations in this work were achieved by using Uncertainty Toolbox v0.1.1 package [17].

N7 Computational cost comparison

For the AdsMT model adopting the AdsGT encoder, it takes approximately 12 seconds on a RTX 3090 GPU to directly predict the GMAE of 1000 surface/adsorbate combinations, resulting in a prediction speed of 0.012 seconds per combination. In terms of the method combining machine learning interatomic potentials (MLIP) with heuristic searches to compute GMAE, we compare AdsMT with the AdsorbML workflow [3]. For a single surface/adsorbate combination, AdsorbML generates 100 initial adsorption structures using heuristic and random methods (num site=100). Each initial structure is then optimized using MLIP. Through the GemNet-OC model [20] with 39M parameters pretrained on the OC20 dataset [4], each 80-step structure relaxation takes about 4 seconds on a RTX 3090 GPU. Therefore, AdsorbML needs about 400 seconds to calculate the GMAE of one surface/adsorbate combination, and the AdsMT model is about 3.3×10^4 times faster than the AdsorbML pipeline. In addition, the AdsorbML process using GemNet-OC-39M accelerates the GMAE calculation by about 4815 times compared to the DFT method [3]. Correspondingly, the AdsMT model is about 1.6×10^8 times faster than DFT methods in GMAE calculations.

Given a target reaction involving two key intermediate adsorbates and a database containing 200,000 catalysts, assuming 20 surface structures are generated from each catalyst, a total of 4,000,000 possible surfaces can be obtained. For the AdsMT model, it would only require around 1 GPU/day to compute the GMAE of all possible surfaces for the two key adsorbates. However, it would spend about 37037 GPU/day using pre-trained machine learning interatomic potentials (GemNet-OC-39M) with AdsorbML pipeline [3]. Even using 100 GPUs simultaneously, it still needs 370 days to complete all calculations. Therefore, the AdsMT model is more cost-effective and holds greater potential for large-scale catalyst pre-screening, especially when dealing with large catalyst databases ($\geq$ 1M).

N8 Hyperparameters for AdsMT models

We optimized the important hyperparameters of AdsMT models by performing a small-scale grid search using the fake subset from OC20-LMAE. The tuned hyperparameters include the number of hidden channels, message passing layer number of graph encoder, layer number of vector encoder, batch size, initial learning rate, learning rate gamma, and max epoch number. The Hyperparameters for the AdsMT models with the AdsGT encoder are provided in Tables S11, S12 and S13. Hyperparameters of the remaining AdsMT models can be found in the corresponding repo: `https://github.com/schwallergroup/AdsMT/tree/main/configs`.

N9 Similarity between OC20-LMAE and GMAE datasets

The similarity between the source and target domains is also important for successful transfer learning. Both OCD-GMAE and OC20-LMAE datasets originate from the Open Catalyst Project [4] with analogous surface and adsorbate types and the same DFT methods, which will be advantageous for transfer learning. The surfaces in both datasets were generated from crystals in the Materials Project database, which includes 54 elements, various crystal systems and space groups, and crystal facets with a maximum Miller index of less than 2. However, the surfaces in Alloy-GMAE were generated from binary alloys with face-centered cubic structures and only used the (111) Miller index. The FG-GMAE dataset contains only 14 different surfaces from pure metals with a face-centered cubic structure. The significant differences in the distributions of surface compositions and geometries between the pre-training dataset and the target GMAE dataset could limit the effectiveness of transfer learning in enhancing AdsMT performance.

N10 Identify different types of adsorption sites from cross-attention scores

Furthermore, we explored extending our method to identify different types of adsorption sites (e.g., top, bridge, hollow) rather than simply assessing the importance of individual surface atoms. Given a set of surface atoms $\mathbf{A}$ and their attention scores $\mathbf{S}$, higher attention scores indicate that the atom is more important for strong adsorption. The maximum attention score is defined as s_{max}, and the set of atoms with scores above $0.8s_{max}$ is defined as the potential site atoms $\mathbf{P}$. For the atom i in $\mathbf{P}$, if no atom j in $\mathbf{P}$ satisfies Equation S6, then atom i is considered to constitute a top site. For the atom i in $\mathbf{P}$, if there is only one atom j in P that satisfies Equation S6, then atoms i and j are considered to constitute a bridge site. If there are three atoms i, j and k in $\mathbf{P}$, where d_{ij}, d_{ik} and d_{jk} all satisfy Equation S6 and form a triangle, then atoms i, j and k are considered to constitute a hollow site.

$$d_{ij} < r_i + r_j + \delta, \quad i \neq j \tag{S6}$$

In Equation S6, d_{ij} represents the distance between atoms i and j, r_i and r_j are the covalent radii of atoms i and j, respectively, and δ can either be a fixed value or the geometric diameter of the adsorbate. We evaluated this extended method using our AdsMT models, without considering more complex site types (e.g., 4-fold hollow site). As shown in Figure S14, based on the improved method described above, it is possible to identify adsorption site types (top, bridge, hollow) using attention scores and the distances between high-scoring atoms. Isolated high-scoring atoms without surrounding high-scoring atoms tend to form top sites, while high-scoring atoms close to each other are more likely to form bridge or hollow sites. The results show that the cross-attention scores have the potential to identify the adsorption site type through the improved method, which will be further studied in future work.

N11 Sensitivity of the AdsMT model to surface geometric fluctuations

To study the sensitivity of the AdsMT model to the surface's geometry (e.g., atom distances and lattice constants), we added noise to the input surface structures without changing atomic connectivity through the following methods:

1) randomly perturbing atomic coordinates in the x, y, and z directions (displacements < 0.2 Å);

2) randomly perturbing the lengths of lattice vectors a and b (deviations $< 2\%$). The c vector, which corresponds to the vacuum layer direction, was unchanged.

By comparing GMAE predictions from the original and noise-perturbed surface structures, we evaluated the prediction fluctuations and mean energy differences induced by geometric variations. As shown in Table S14, AdsMT models with different graph encoders exhibit low energy prediction fluctuation after adding geometric noise to the input surfaces. In particular on the FG-GMAE dataset, the mean absolute energy differences of GMAE predictions between original and noise-perturbed surfaces are very small, only about 0.014 eV. The results show that our model is insensitive to geometric variations of input surfaces where atomic connectivity does not change. This insensitivity is advantageous for AdsMT models in virtual catalyst screening, where surface structures could be generated from different crystal databases.

Supplementary Figures

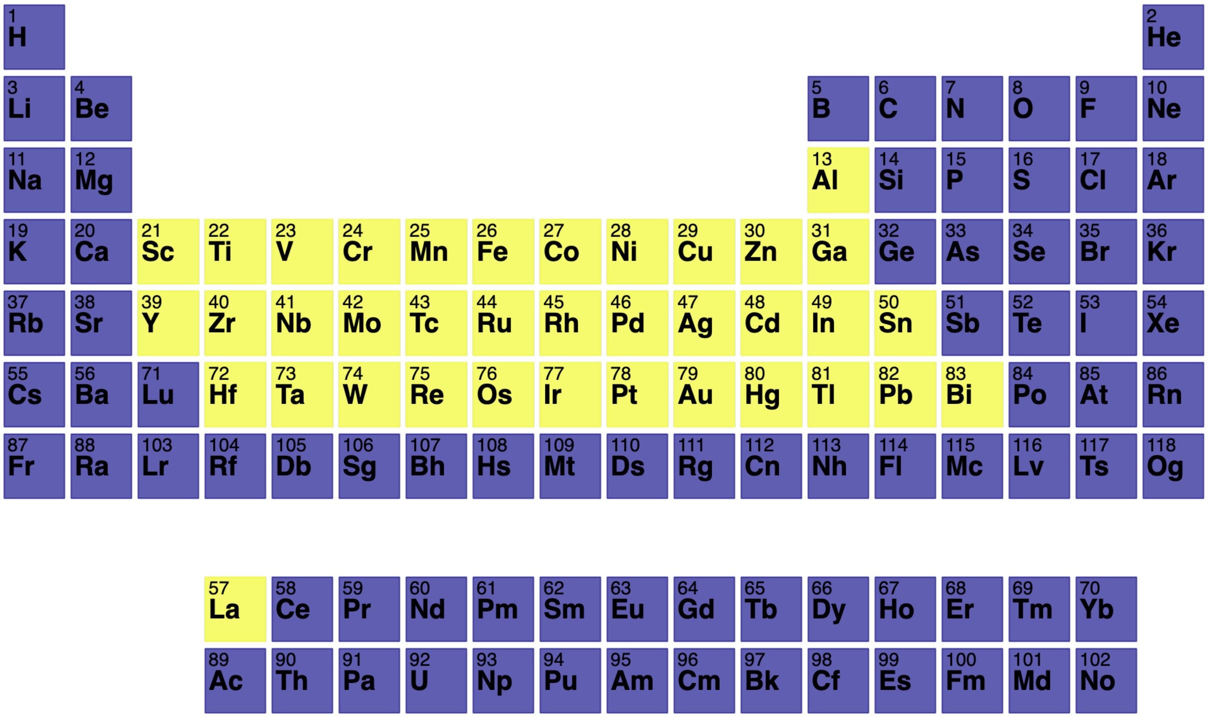

Figure S1: The periodic table outlining elements included in the Alloy-GMAE dataset.

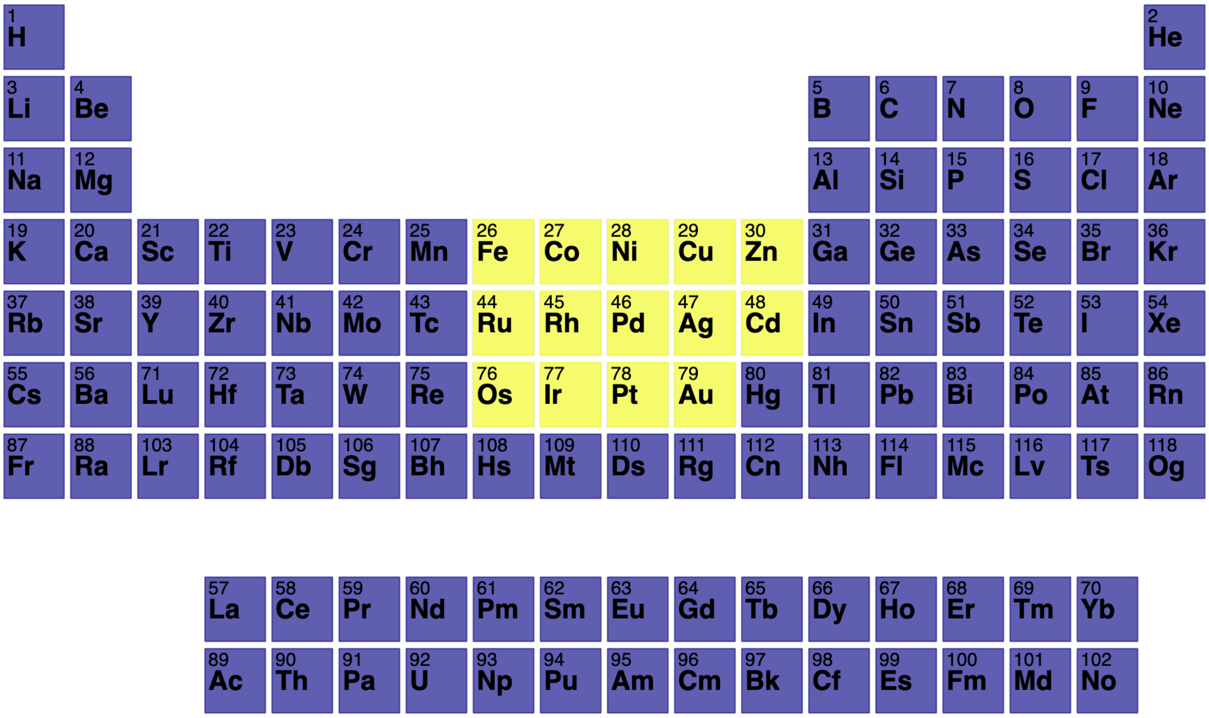

Figure S2: The periodic table outlining elements included in the FG-GMAE dataset.

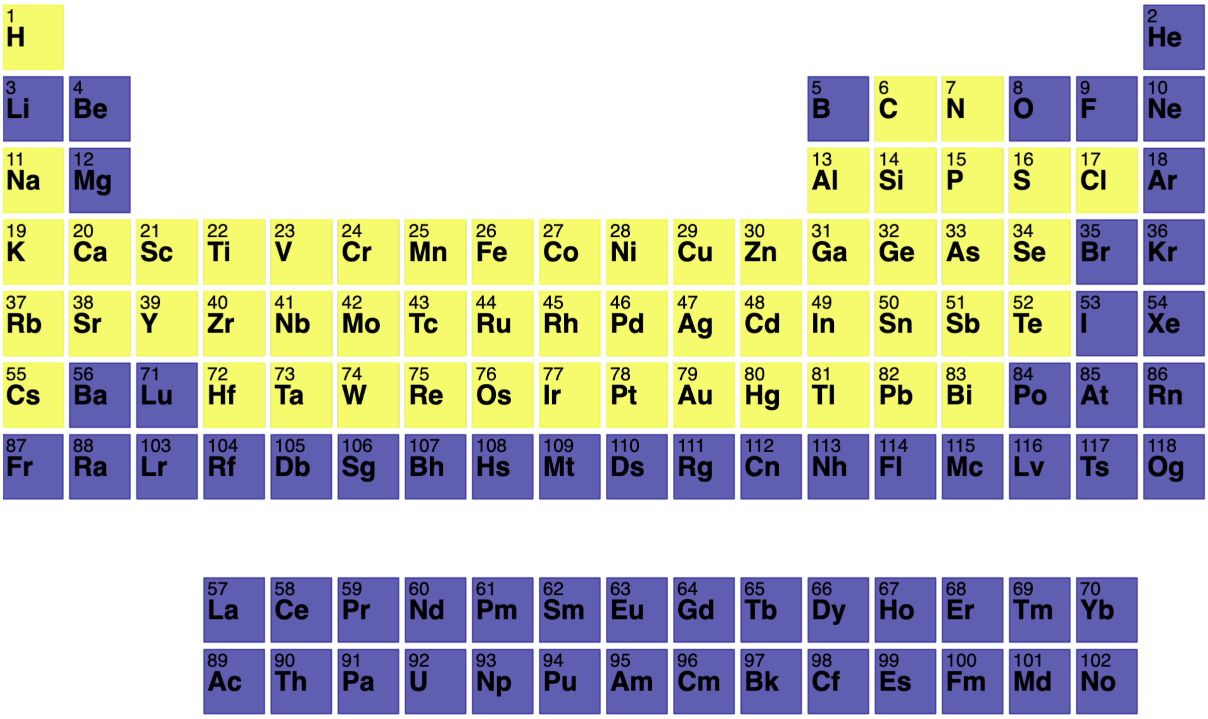

Figure S3: The periodic table outlining elements included in the OCD-GMAE dataset.

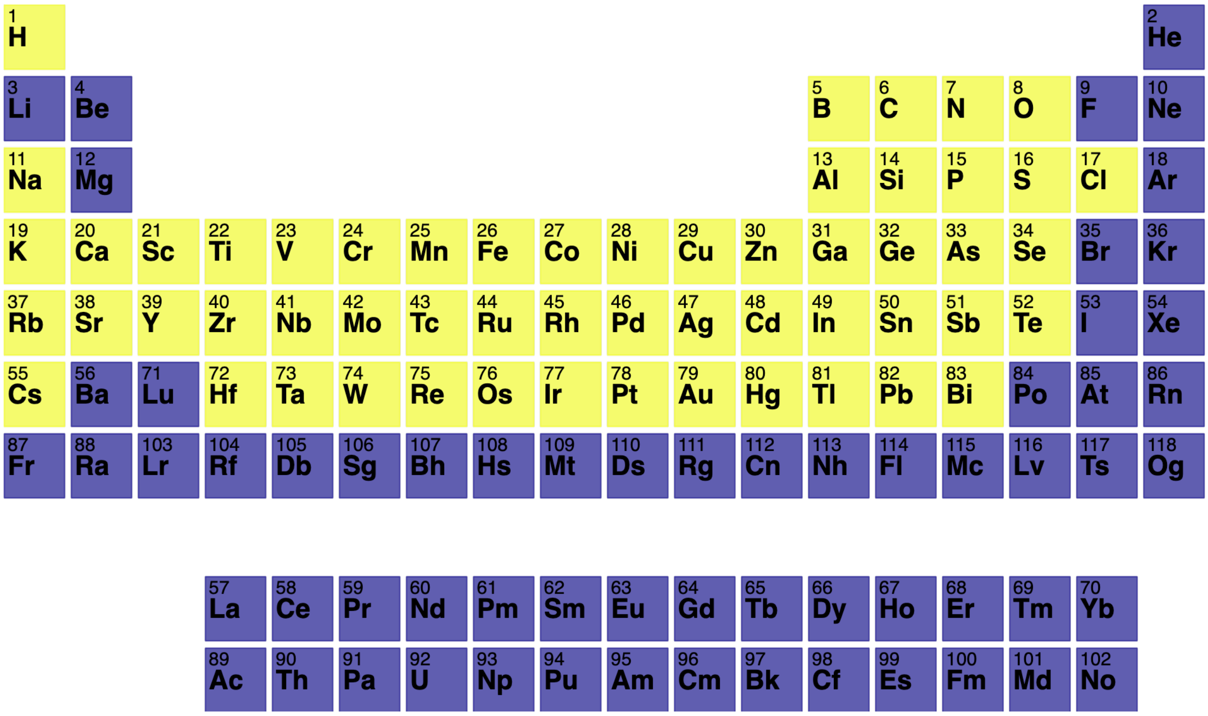

Figure S4: The periodic table outlining elements included in the OC20-LMAE dataset.

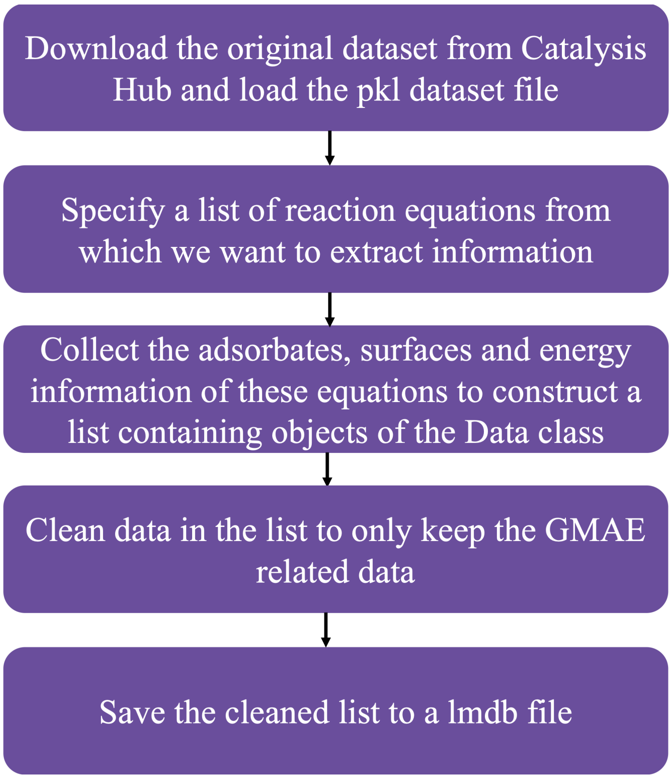

Figure S5: A brief illustration of the Alloy-GMAE dataset construction process.

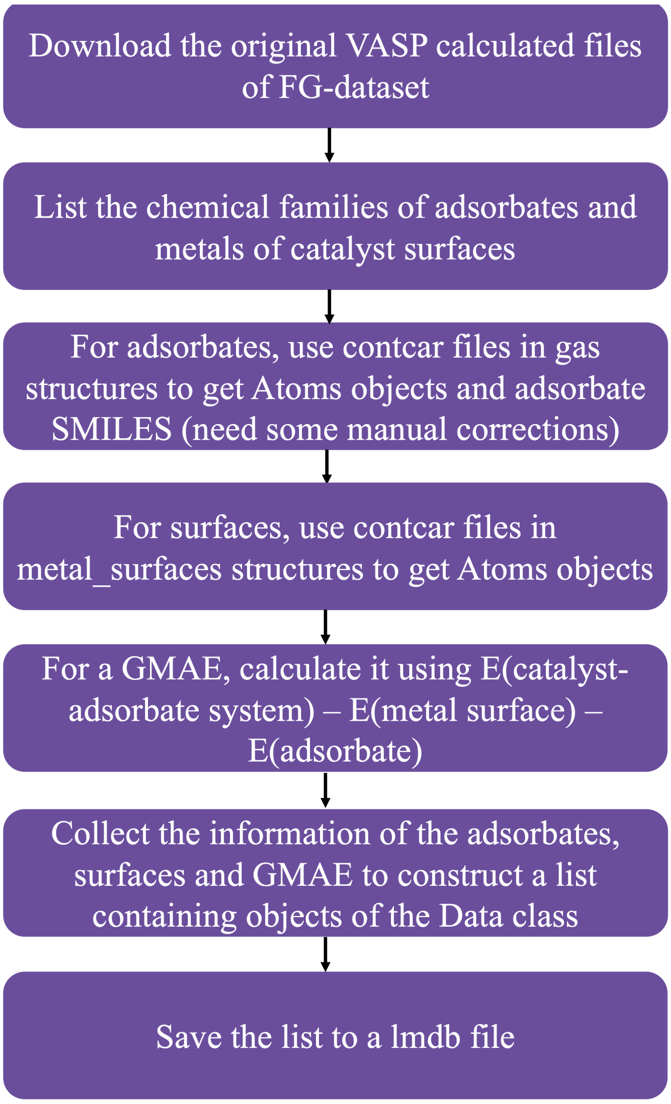

Figure S6: A brief illustration of the FG-GMAE dataset construction process.

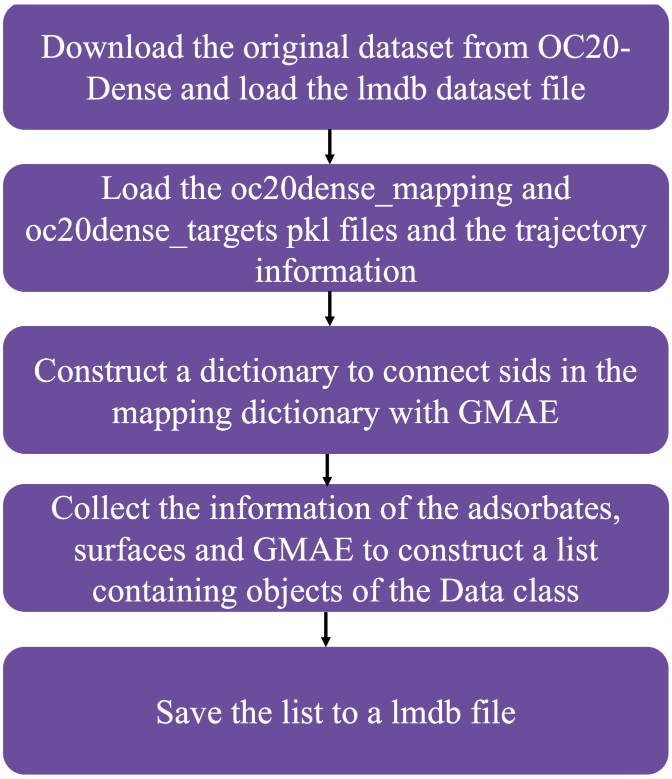

Figure S7: A brief illustration of the OCD-GMAE dataset construction process.

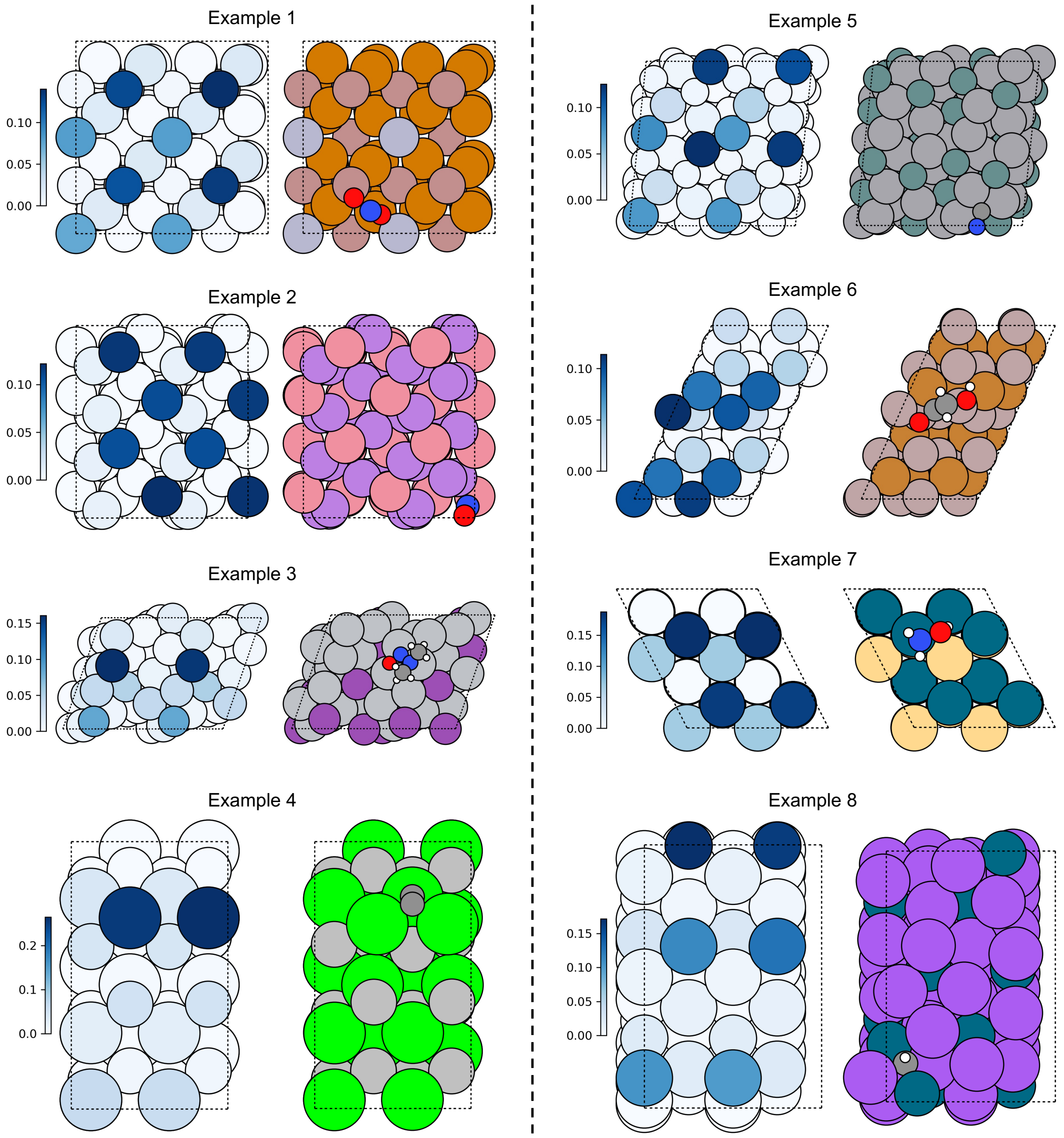

Figure S8: Eight examples comparing top views of cross-attention score-colored surfaces (left) and global minimum adsorption structures optimized by density functional theory (right), where the cross-attention scores are computed by the trained AdsMT model adopting GemNet-OC graph encoder. The color bar depicts the value of the cross-attention score of the surface atoms.

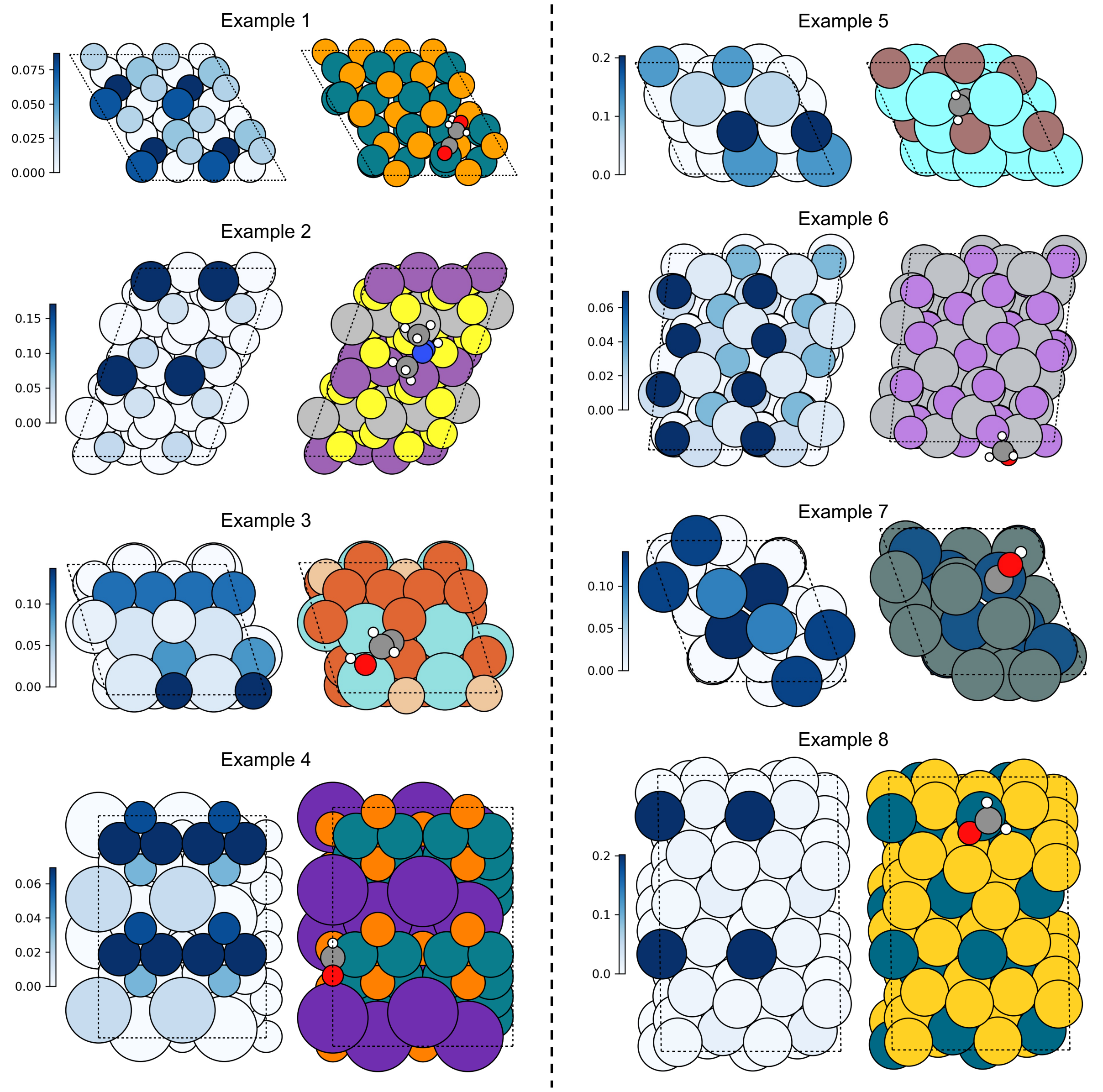

Figure S9: Eight examples comparing top views of cross-attention score-colored surfaces (left) and global minimum adsorption structures optimized by density functional theory (right), where the cross-attention scores are computed by the trained AdsMT model adopting AdsGT graph encoder. The color bar depicts the value of the cross-attention score of the surface atoms.

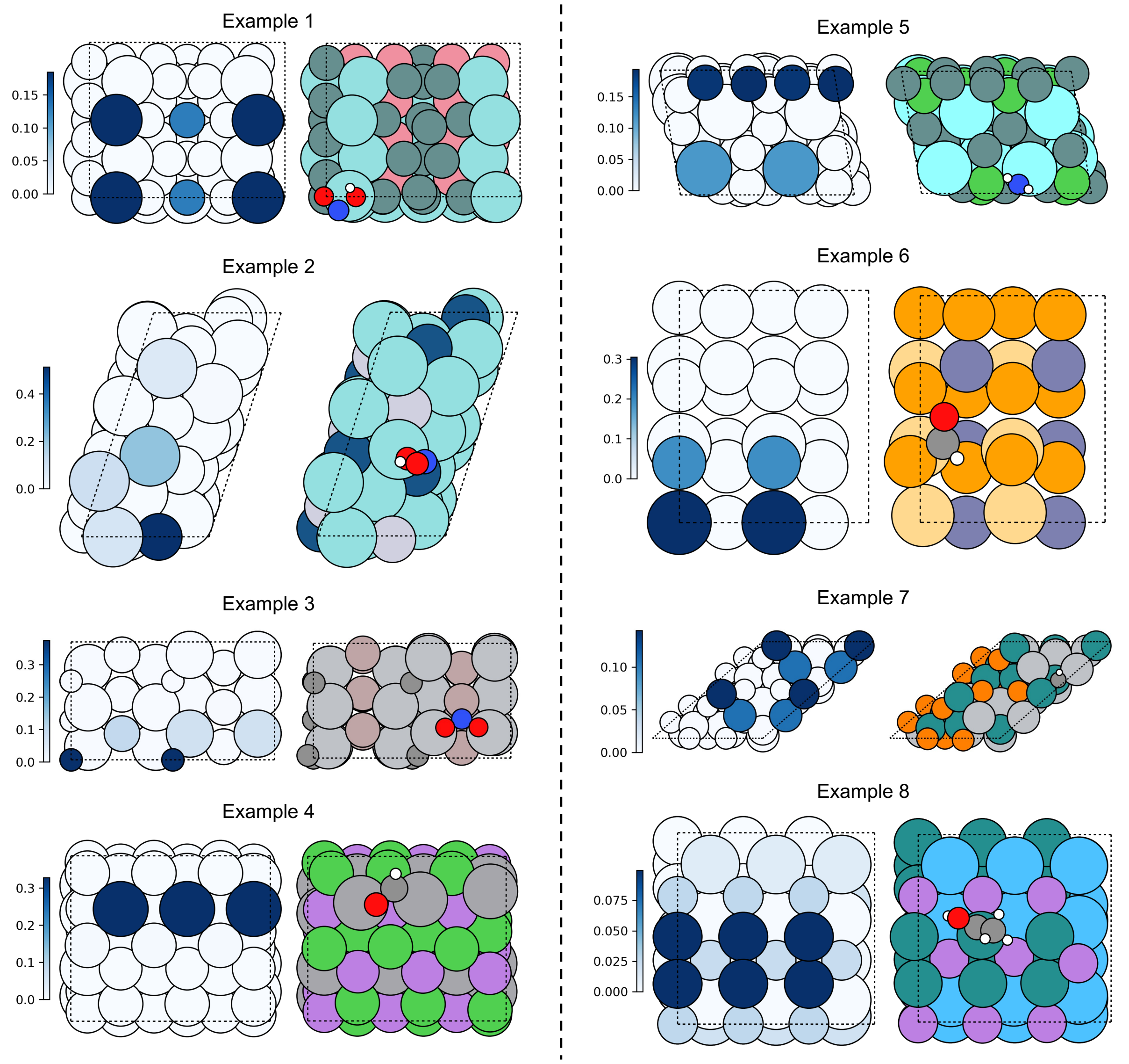

Figure S10: Eight examples comparing top views of cross-attention score-colored surfaces (left) and global minimum adsorption structures optimized by density functional theory (right), where the cross-attention scores are computed by the trained AdsMT model adopting eSCN graph encoder. The color bar depicts the value of the cross-attention score of the surface atoms.

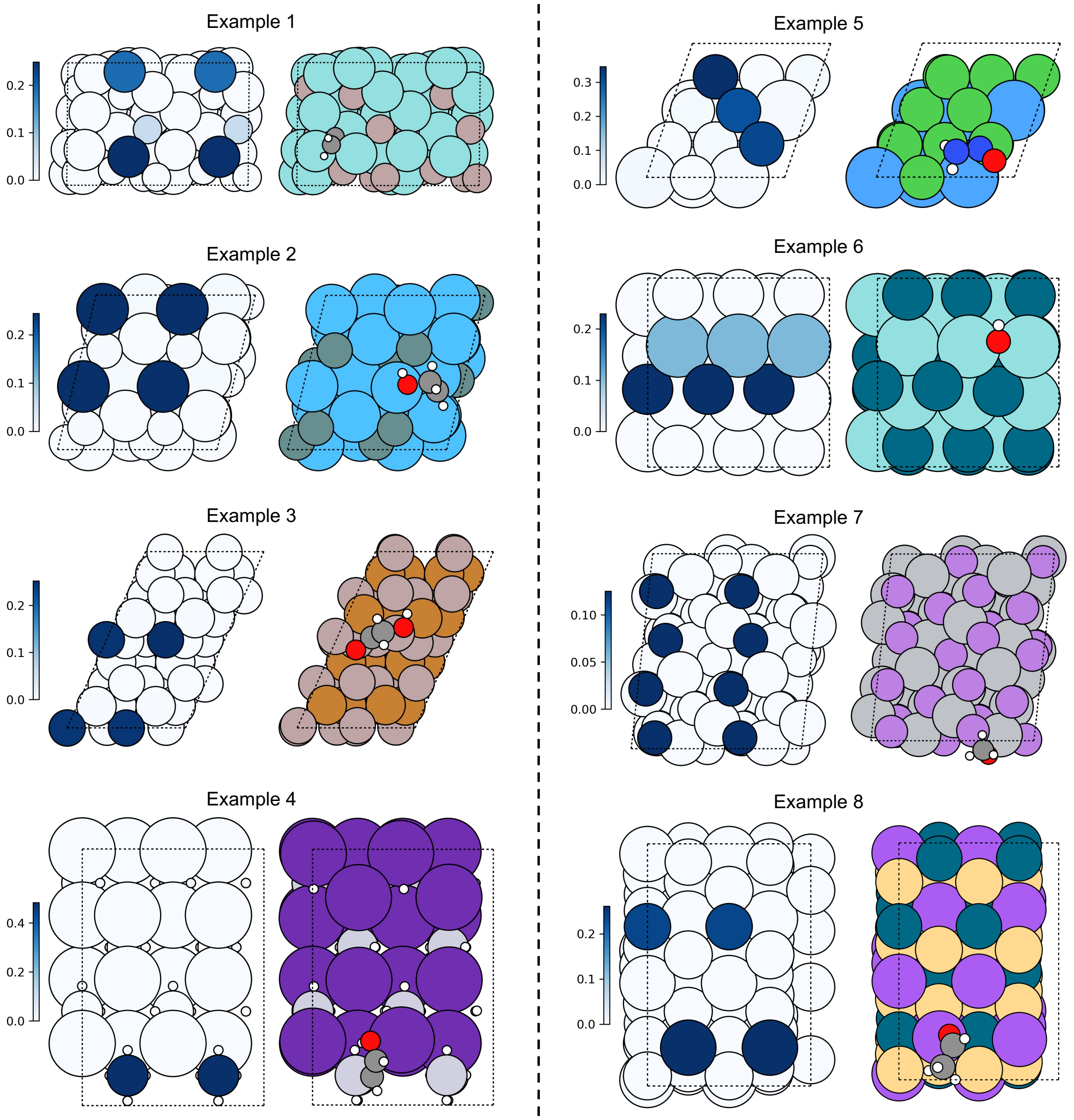

Figure S11: Eight examples comparing top views of cross-attention score-colored surfaces (left) and global minimum adsorption structures optimized by density functional theory (right), where the cross-attention scores are computed by the trained AdsMT model adopting ET graph encoder. The color bar depicts the value of the cross-attention score of the surface atoms.

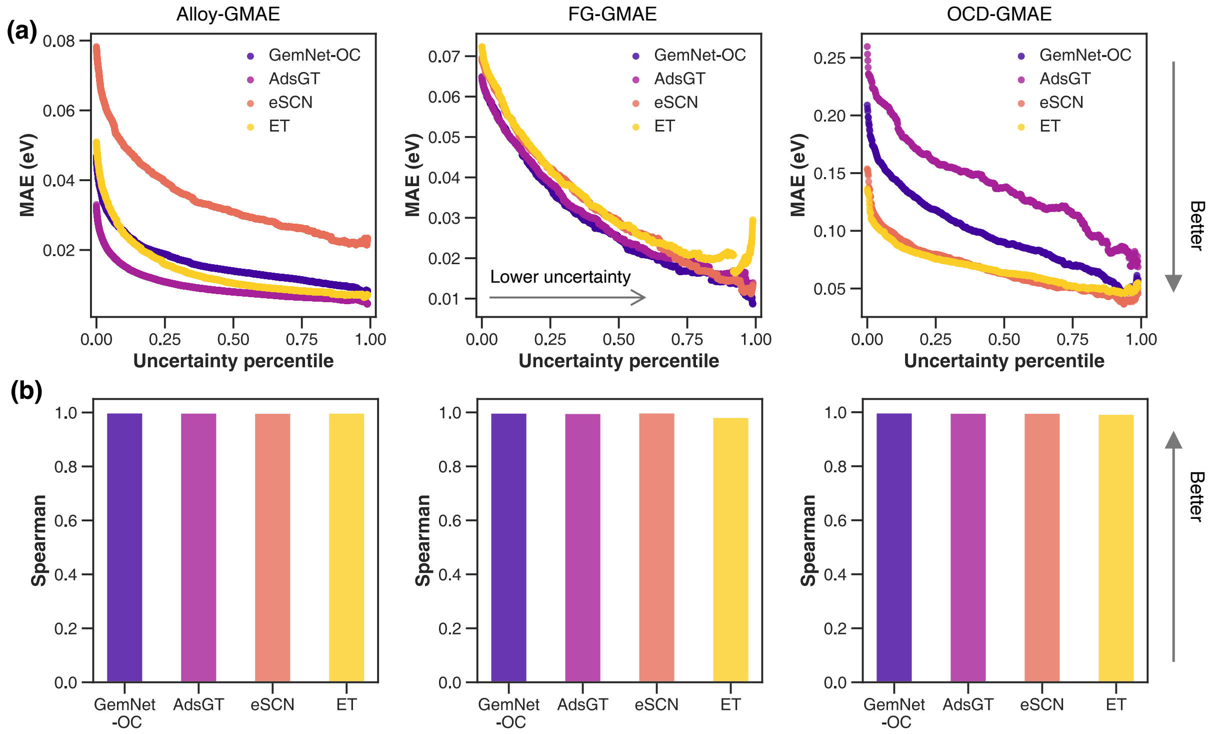

Figure S12: Uncertainty estimation of AdsMT models with different graph encoders on the GMAE datasets: (a) Cumulative MAE at different cutoffs of uncertainty percentiles, and corresponding (b) Spearman correlation coefficients between the estimated uncertainty and cumulative MAE.

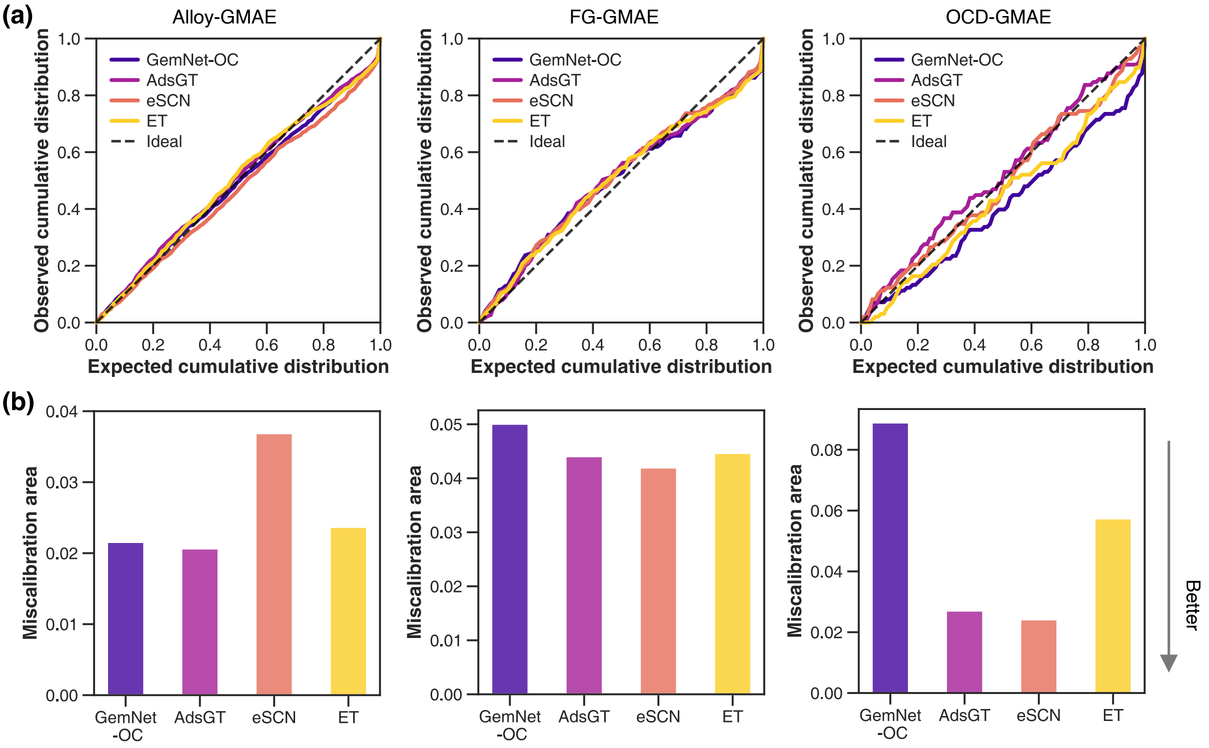

Figure S13: Calibration curves (a) of AdsMT models with different graph encoders after recalibration on the GMAE datasets, and corresponding (b) miscalibration area.

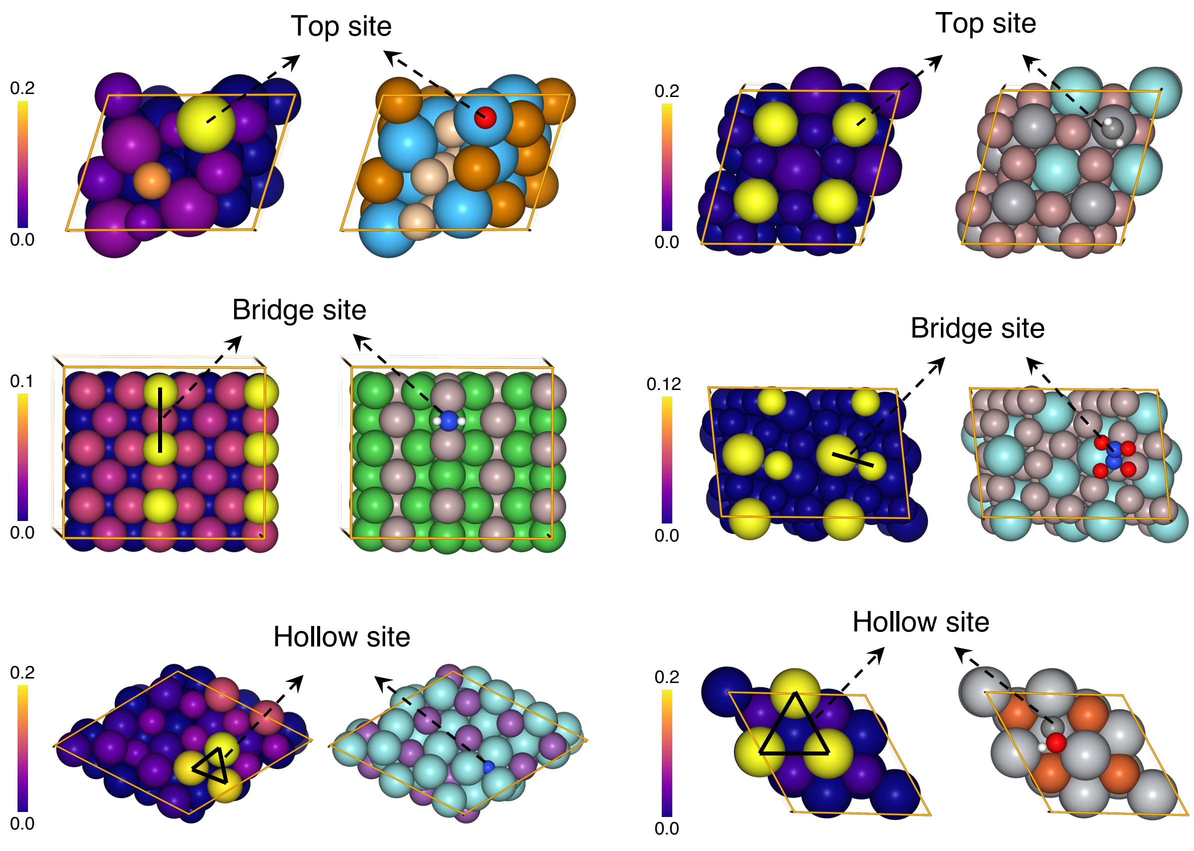

Figure S14: Examples of identifying different adsorption site types based on the extended method and cross-attention scores.

Left: cross-attention score-colored surfaces. Right: global minimum adsorption structures optimized by density functional theory.

Supplementary Tables

Table S1: Overview of three new GMAE datasets.

Dataset	Combination Num.	Surface Num.	Adsorbate Num.	Range of GMAE (eV)
OCD-GMAE	973	967 (54)	74 (4)	-8.0 ~ 6.4
Alloy-GMAE	11,260	1,916 (37)	12 (5)	-4.3 ~ 9.1
FG-GMAE	3,308	14 (14)	202 (5)	-4.0 ~ 0.8

[1] () values represent the numbers of element types.

Table S2: Adsorbate information for Alloy-GMAE dataset

Adsorbate class	Adsorbates Num.	Adsorbate
only contain O/H	4	*H, *O, *OH, *H_2O
C_1	4	*C, *CH, *CH_2, *CH_3
Sulfur-based	2	*S, *SH
Nitrogen-based	2	*N, *NH

Table S3: Adsorbate information for FG-GMAE dataset

Chemical family	Adsorbates Num.
Hydrocarbons, alcohols, aldehydes, ketones and ethers	32
Carbonates, carboxylic acids, and esters	18
Amines and imines	32
Amidines	40
Thiols, thials, thioketones and thioethers	19
Amides	15
Oximes	12
Carbamate esters	15
Aromatic molecules	24

Table S4: Adsorbate information for OCD-GMAE dataset

Adsorbate class	Adsorbate Num.	Adsorbate
O/H only	4	*H, *O, *OH, *OH_2
C_1	12	*C, *CH, *CHO, *COH, *CH_2, *CH_2*O, *CHOH, *CH_3, *OCH_3, *CH_2OH, *CH_4, *$OHCH_3$
C_2	37	*C*C, *CCO, *CCH, *CHCO, *CCHO, *COCHO, *CCHOH, *CCH_2, *CH*CH, CH_2*CO, *CHCHO, *$COCH_2O$, *CHO*CHO, *COHCHO, *COHCOH, *CCH_3, *$CHCH_2$, *$COCH_3$, *CHCHOH, *CCH_2OH, *CHOCHOH, *$COCH_2OH$, *COHCHOH, *$OCHCH_3$, *$COHCH_3$, *$CHOHCH_2$, *$CHCH_2OH$, *OCH_2CHOH, *$CHOCH_2OH$, *$COHCH_2OH$, *CHOHCHOH, *CH_2CH_3, *OCH_2CH_3, *$CHOHCH_3$, *CH_2CH_2OH, *$CHOHCH_2OH$, *$OHCH_2CH_3$
Nitrogen-based	21	*$NH_2N(CH_3)_2$, *$ONN(CH_3)_2$, *$OHNNCH_3$, *ONH, *NHNH, *N*NH, *NO_2NO_2, *N*NO, *N_2, *$ONNH_2$, *NH_2, *NH_3, *NONH, *NH, *NO_2, *NO, *N, *NO_3, *$OHNH_2$, *ONOH, *CN

Table S5: List of adsorbate descriptors used in the AdsMT model

Descriptor/Descriptor Family	Notes
Gasteiger/Marsili Partial Charges	*Tetrahedron* **36**:3219–28 (1980)
BalabanJ	*Chem. Phys. Lett.* **89**:399–404 (1982)
BertzCT	*J. Am. Chem. Soc.* **103**:3599–601 (1981)
Ipc	*J. Chem. Phys.* **67**:4517–33 (1977)
HallKierAlpha	*Rev. Comput. Chem.* **2**:367–422 (1991)
Kappa1 – Kappa3	*Rev. Comput. Chem.* **2**:367–422 (1991)
Phi	New in 2021.03 release *Quant. Struct.–Act. Rel.* **8**:221–224 (1989)
Chi0, Chi1	*Rev. Comput. Chem.* **2**:367–422 (1991)
Chi0n – Chi4n	*Rev. Comput. Chem.* **2**:367–422 (1991)
Chi0v – Chi4v	*Rev. Comput. Chem.* **2**:367–422 (1991)
MolLogP	Wildman and Crippen *JCICS* **39**:868–73 (1999)
MolMR	Wildman and Crippen *JCICS* **39**:868–73 (1999)
MolWt	Average molecular weight of the molecule
ExactMolWt	Exact molecular weight of the molecule
HeavyAtomCount	Number of heavy atoms in a molecule
HeavyAtomMolWt	Average molecular weight of the molecule ignoring hydrogens
NHOHCount	Number of NHs or OHs
NOCount	Number of nitrogens and oxygens
NumHAcceptors	Number of hydrogen bond acceptors
NumHDonors	Number of hydrogen bond donors
NumHeteroatoms	Number of heteroatoms
NumRotatableBonds	Number of rotatable bonds
NumValenceElectrons	Number of valence electrons the molecule has
NumAmideBonds	Number of amide bonds
Num{Aromatic,Saturated, Aliphatic}Rings	Number of aromatic/ saturated/ aliphatic(at least one non-aromatic bond) rings in a molecule
Num{Aromatic,Saturated, Aliphatic}{Hetero,Carbo}cycles	Number of aromatic/ saturated/ aliphatic(at least one non-aromatic bond) heterocycles/ carbocycles in a molecule

Table S5: (continued)

Descriptor/Descriptor Family	Notes
RingCount	Number of rings in a molecule
FractionCSP3	Fraction of carbons that are sp3 hybridized
NumSpiroAtoms	Number of spiro atoms (atoms shared between rings that share exactly one atom)
NumBridgeheadAtoms	Number of bridgehead atoms (atoms shared between rings that share at least two bonds)
TPSA	*J. Med. Chem.* **43**:3714–7, (2000) See the section in the RDKit book describing differences to the original publication.
LabuteASA	*J. Mol. Graph. Mod.* **18**:464–77 (2000)
PEOE_VSA1 – PEOE_VSA14	MOE-type descriptors using partial charges and surface area contributions http://www.chemcomp.com/journal/vsadesc.htm
SMR_VSA1 – SMR_VSA10	MOE-type descriptors using MR contributions and surface area contributions http://www.chemcomp.com/journal/vsade-sc.htm
SlogP_VSA1 – SlogP_VSA12	MOE-type descriptors using LogP contributions and surface area contributions http://www.chemcomp.com/journal/vsade-sc.htm
EState_VSA1 – EState_VSA11	MOE-type descriptors using EState indices and surface area contributions (developed at RD, not described in the CCG paper)
VSA_EState1 – VSA_EState10	MOE-type descriptors using EState indices and surface area contributions (developed at RD, not described in the CCG paper)
MQNs	Nguyen et al. *ChemMedChem* **4**:1803–5 (2009)
Topliss fragments	implemented using a set of SMARTS definitions in $(RDBASE)/Data/FragmentDescriptors.csv

Table S5: (continued)

Descriptor/Descriptor Family	Notes
Autocorr2D	New in 2017.09 release. Todeschini and Consoni "Descriptors from Molecular Geometry" Handbook of Chemoinformatics https://doi.org/10.1002/9783527618279.ch37
BCUT2D	New in 2020.09 release. Pearlman and Smith in "3D–QSAR and Drug Design: Recent Advances" (1997)

Table S6: Test MAE results (eV) of AdsMT framework with different graph encoders on the three GMAE datasets without transfer learning. The best results for each GMAE dataset are marked in bold.

Graph encoders	Alloy-GMAE	FG-GMAE	OCD-GMAE
CGCNN	0.159 ± 0.012	0.110 ± 0.010	0.652 ± 0.066
SchNet	0.162 ± 0.010	0.103± 0.012	0.661 ± 0.056
DimeNet++	0.152 ± 0.010	0.107 ± 0.013	0.671 ± 0.073
GemNet-OC	0.146 ± 0.010	0.097 ± 0.007	0.645 ± 0.048
ET	0.146 ± 0.009	0.098 ± 0.008	0.686 ± 0.065
eSCN	0.163 ± 0.011	0.102 ± 0.009	0.589 ± 0.046
AdsGT (this work)	**0.143** ± **0.008**	**0.095** ± **0.007**	**0.571** ± **0.057**

Table S7: Test success rate results (%) of AdsMT framework with different graph encoders on the three GMAE datasets without transfer learning. The best results for each GMAE dataset are marked in bold.

Graph encoders	Alloy-GMAE	FG-GMAE	OCD-GMAE
CGCNN	59.7 ± 2.5	67.1 ± 2.7	11.7 ± 4.2
SchNet	57.7 ± 1.7	68.9 ± 3.6	10.6 ± 2.6
DimeNet++	61.3 ± 2.6	67.3 ± 4.6	11.0 ± 4.5
GemNet-OC	65.0 ± 1.6	71.2 ± 2.3	12.5 ± 2.3
ET	64.3 ± 1.7	71.6 ± 2.6	11.9 ± 2.9
eSCN	56.8 ± 2.0	69.8 ± 2.9	12.8 ± 2.0
AdsGT (this work)	**66.3 ± 1.3**	**71.9 ± 2.4**	**13.5 ± 4.4**

Table S8: Performance comparison of MAE (unit: eV) and success rate (SR, unit: %) for AdsMT models adopting different graph encoders w/o and with transfer learning (TL) on the Alloy-GMAE dataset.

	GemNet-OC	ET	eSCN	AdsGT
MAE w/o TL	0.146 ± 0.010	0.146 ± 0.009	0.163 ± 0.011	0.143 ± 0.008
MAE with TL	0.141 ± 0.011	0.142 ± 0.009	0.153 ± 0.006	0.140 ± 0.010
MAE decrease	0.005	0.004	0.010	0.003
SR w/o TL	65.0 ± 1.6	64.3 ± 1.7	56.8 ± 2.0	66.3 ± 1.3
SR with TL	66.5 ± 1.5	67.0 ± 1.1	60.8 ± 1.1	68.4 ± 0.8
SR increase	1.5	2.7	4.0	2.1

Table S9: Performance comparison of MAE (unit: eV) and success rate (SR, unit: %) for AdsMT models adopting different graph encoders w/o and with transfer learning (TL) on the FG-GMAE dataset.

	GemNet-OC	ET	eSCN	AdsGT
MAE w/o TL	0.097 ± 0.007	0.098 ± 0.008	0.102 ± 0.009	0.095 ± 0.007
MAE with TL	0.095 ± 0.008	0.097 ± 0.007	0.096 ± 0.007	0.094 ± 0.008
MAE decrease	0.002	0.001	0.006	0.001
SR w/o TL	71.2 ± 2.3	71.6 ± 2.6	69.8 ± 2.9	71.9 ± 2.4
SR with TL	72.3 ± 2.3	72.0 ± 2.1	72.2 ± 2.7	72.6 ± 1.8
SR increase	1.1	0.4	2.4	0.7

Table S10: Performance comparison of MAE (unit: eV) and success rate (SR, unit: %) for AdsMT models adopting different graph encoders w/o and with transfer learning (TL) on the OCD-GMAE dataset.

	GemNet-OC	ET	eSCN	AdsGT
MAE w/o TL	0.645 ± 0.048	0.686 ± 0.065	0.589 ± 0.046	0.571 ± 0.057
MAE with TL	0.389 ± 0.048	0.395 ± 0.043	0.406 ± 0.037	0.430 ± 0.052
MAE decrease	0.256	0.291	0.183	0.141
SR w/o TL	12.5 ± 2.3	11.9 ± 2.9	12.8 ± 2.0	13.5 ± 4.4
SR with TL	22.0 ± 4.7	21.2 ± 3.3	21.0 ± 4.2	21.4 ± 3.6
SR increase	9.5	9.3	8.2	7.9

Table S11: Hyperparameters for the AdsMT model with AdsGT graph encoder on the Alloy-GMAE dataset.

Graph encoder	
Node embedding dimension	128
Edge embedding dimension	128
Number of attention layers	3
Number of attention heads	8
Cutoff of edge definition	8.0 Å
max neighbors	12
Vector encoder	
Hidden dimension	128
Number of Linear layers	2
Cross-modal encoder	
Atom embedding dimension	128
Vector embedding dimension	128
Number of Gaussians for smearing	100
Number of cross-attention heads	4
Number of self-attention heads	4
Number of attention layers	1
Number of MLP layers for energy	3
MLP hidden dimension for energy	256
Activation function	SiLU
Training conditions	
Batch size	64
Initial learning rate	0.001
Scheduler	ReduceLROnPlateau
Learning rate factor	0.7
Learning rate patience	10
Max epochs	200

Table S12: Hyperparameters for the AdsMT model with AdsGT graph encoder on the FG-GMAE dataset.

Graph encoder	
Node embedding dimension	128
Edge embedding dimension	128
Number of attention layers	5
Number of attention heads	8
Cutoff of edge definition	6.0 Å
max neighbors	50
Vector encoder	
Hidden dimension	128
Number of Linear layers	2
Cross-modal encoder	
Atom embedding dimension	128
Vector embedding dimension	128
Number of Gaussians for smearing	20
Number of cross-attention heads	4
Number of self-attention heads	4
Number of attention layers	1
Number of MLP layers for energy	3
MLP hidden dimension for energy	256
Activation function	SiLU
Training conditions	
Batch size	16
Initial learning rate	0.0005
Scheduler	ReduceLROnPlateau
Learning rate factor	0.6
Learning rate patience	3
Max epochs	80

Table S13: Hyperparameters for the AdsMT model with AdsGT graph encoder on the OCD-GMAE dataset.

Graph encoder	
Node embedding dimension	128
Edge embedding dimension	128
Number of attention layers	5
Number of attention heads	8
Cutoff of edge definition	6.0 Å
max neighbors	50
Vector encoder	
Hidden dimension	128
Number of Linear layers	2
Cross-modal encoder	
Atom embedding dimension	128
Vector embedding dimension	128
Number of Gaussians for smearing	20
Number of cross-attention heads	4
Number of self-attention heads	4
Number of attention layers	1
Number of MLP layers for energy	3
MLP hidden dimension for energy	256
Activation function	SiLU
Training conditions	
Batch size	8
Initial learning rate	0.0005
Scheduler	ReduceLROnPlateau
Learning rate factor	0.6
Learning rate patience	3
Max epochs	60

Table S14: Mean absolute energy differences (eV) of GMAE predictions between original and noise-perturbed surface structures using the same trained models.

Graph encoders	Alloy-GMAE	FG-GMAE	OCD-GMAE
SchNet	0.053	0.016	0.077
CGCNN	0.047	0.011	0.068
DimNet++	0.068	0.021	0.073
GemNet-OC	0.054	0.016	0.046
ET	0.044	0.014	0.054
eSCN	0.028	0.009	0.045
AdsGT	0.031	0.012	0.052
**Average**	0.046	0.014	0.059

Table S15: Test MAE (eV) results of AdsMT models using different dataset splitting methods on the Alloy-GMAE dataset

Split method	AdsMT (SchNet)	AdsMT (eSCN)	AdsMT (GemNet-OC)	AdsMT (ET)	AdsMT (AdsGT)
Random	0.162 ± 0.010	0.163 ± 0.011	0.146 ± 0.010	0.146 ± 0.009	0.143 ± 0.008
Surface type	0.193 ± 0.015	0.176 ± 0.014	0.165 ± 0.018	0.161 ± 0.013	0.158 ± 0.014
	0.031	0.013	0.019	0.015	0.015

Table S16: Test success rate (SR, %) results of AdsMT models using different dataset splitting methods on the Alloy-GMAE dataset.

Split method	AdsMT (SchNet)	AdsMT (eSCN)	AdsMT (GemNet-OC)	AdsMT (ET)	AdsMT (AdsGT)
Random	57.7 ± 1.7	56.8 ± 2.0	65.0 ± 1.6	64.3 ± 1.7	66.3 ± 1.3
Surface type	48.5 ± 1.9	51.2 ± 1.8	57.2 ± 2.5	58.9 ± 1.7	60.1 ± 2.0
	9.2	5.6	7.8	5.4	6.2

Table S17: Test MAE (eV) results of AdsMT models using different dataset splitting methods on the FG-GMAE dataset.

Split method	AdsMT (SchNet)	AdsMT (eSCN)	AdsMT (GemNet-OC)	AdsMT (ET)	AdsMT (AdsGT)
Random	0.103 ± 0.012	0.102 ± 0.009	0.097 ± 0.007	0.098 ± 0.008	0.095 ± 0.007
Adsorbate type	0.140 ± 0.031	0.147 ± 0.036	0.138 ± 0.038	0.127 ± 0.026	0.123 ± 0.018
	0.037	0.045	0.041	0.029	0.028

Table S18: Test success rate (SR, %) results of AdsMT models using different dataset splitting methods on the FG-GMAE dataset.

Split method	AdsMT (SchNet)	AdsMT (eSCN)	AdsMT (GemNet-OC)	AdsMT (ET)	AdsMT (AdsGT)
Random	68.9 ± 3.6	69.8 ± 2.9	71.2 ± 2.3	71.6 ± 2.6	71.9 ± 2.4
Adsorbate type	59.1 ± 5.3	58.2 ± 5.7	61.4 ± 4.8	64.3 ± 4.3	65.3 ± 3.8
	9.8	11.6	9.8	7.3	6.6

Table S19: Comparison of the success rate (%) and computational speed (comb/min) of AdsMT and pretrained Uni-Mol+ on the OCD-GMAE dataset.

Models	Uni-Mol+				AdsMT after TL
Initial structures	10	25	50	100	—
Success rate (%)	14.8	20.6	29.8	32.7	22.0
Speed (comb./min)	107.1	42.8	21.4	10.7	4902.4

References

[1] Osman Mamun, Kirsten T. Winther, Jacob R. Boes, and Thomas Bligaard. High-throughput calculations of catalytic properties of bimetallic alloy surfaces. *Scientific Data*, 6:76, 2019.

[2] Sergio Pablo-García, Santiago Morandi, Rodrigo A. Vargas-Hernández, Kjell Jorner, Žarko Ivković, Núria López, and Alán Aspuru-Guzik. Fast evaluation of the adsorption energy of organic molecules on metals via graph neural networks. *Nature Computational Science*, 3:433–442, 2023.

[3] Janice Lan, Aini Palizhati, Muhammed Shuaibi, Brandon M Wood, Brook Wander, Abhishek Das, Matt Uyttendaele, C Lawrence Zitnick, and Zachary W Ulissi. Adsorbml: a leap in efficiency for adsorption energy calculations using generalizable machine learning potentials. *npj Computational Materials*, 9(1):172, 2023.

[4] Lowik Chanussot, Abhishek Das, Siddharth Goyal, Thibaut Lavril, Muhammed Shuaibi, Morgane Riviere, Kevin Tran, Javier Heras-Domingo, Caleb Ho, Weihua Hu, Aini Palizhati, Anuroop Sriram, Brandon Wood, Junwoong Yoon, Devi Parikh, C. Lawrence Zitnick, and Zachary Ulissi. Open Catalyst 2020 (OC20) Dataset and Community Challenges. *ACS Catalysis*, 11(10):6059–6072, 2021.

[5] Leland McInnes, John Healy, and James Melville. Umap: Uniform manifold approximation and projection for dimension reduction. *arXiv preprint arXiv:1802.03426*, 2018.

[6] Mclnnes Leland, Healy John, Saul Nathaniel, and Großberger Lukas. Umap: uniform manifold approximation and projection. *Journal of Open Source Software*, 3(29):861, 2018.

[7] Albert P Bartók, Risi Kondor, and Gábor Csányi. On representing chemical environments. *Physical Review B*, 87(18):184115, 2013.

[8] Sandip De, Albert P Bartók, Gábor Csányi, and Michele Ceriotti. Comparing molecules and solids across structural and alchemical space. *Physical Chemistry Chemical Physics*, 18(20):13754–13769, 2016.

[9] Greg Landrum, Paolo Tosco, Brian Kelley, Ric, sriniker, gedeck, Riccardo Vianello, David Cosgrove, NadineSchneider, Eisuke Kawashima, Dan N, Andrew Dalke, Gareth Jones, Brian Cole, Matt Swain, Samo Turk, AlexanderSavelyev, Alain Vaucher, Maciej Wójcikowski, Ichiru Take, Daniel Probst, Kazuya Ujihara, Vincent F. Scalfani, guillaume godin, Axel Pahl, Francois Berenger, JLVarjo, strets123, JP, and DoliathGavid. Rdkit: A software suite for cheminformatics, computational chemistry, and predictive modeling. In *Version: release_2022_09_5*, 2022.

[10] Lauri Himanen, Marc O. J. Jäger, Eiaki V. Morooka, Filippo Federici Canova, Yashasvi S. Ranawat, David Z. Gao, Patrick Rinke, and Adam S. Foster. DScribe: Library of descriptors for machine learning in materials science. *Computer Physics Communications*, 247:106949, 2020.

[11] Jarno Laakso, Lauri Himanen, Henrietta Homm, Eiaki V Morooka, Marc OJ Jäger, Milica Todorović, and Patrick Rinke. Updates to the dscribe library: New descriptors and derivatives. *The Journal of Chemical Physics*, 158(23), 2023.

[12] Yunan Luo, Yang Liu, and Jian Peng. Calibrated geometric deep learning improves kinase–drug binding predictions. *Nature Machine Intelligence*, 5(12):1390–1401, 2023.

[13] Arsenii Ashukha, Alexander Lyzhov, Dmitry Molchanov, and Dmitry P. Vetrov. Pitfalls of in-domain uncertainty estimation and ensembling in deep learning. In *8th International Conference on Learning Representations*, 2020.

[14] Volodymyr Kuleshov, Nathan Fenner, and Stefano Ermon. Accurate uncertainties for deep learning using calibrated regression. In *Proceedings of the 35th International Conference on Machine Learning*, volume 80, pages 2801–2809. PMLR, 2018.

[15] Gabriele Scalia, Colin A Grambow, Barbara Pernici, Yi-Pei Li, and William H Green. Evaluating scalable uncertainty estimation methods for deep learning-based molecular property prediction. *Journal of chemical information and modeling*, 60(6):2697–2717, 2020.

[16] Kevin Tran, Willie Neiswanger, Junwoong Yoon, Qingyang Zhang, Eric Xing, and Zachary W Ulissi. Methods for comparing uncertainty quantifications for material property predictions. *Machine Learning: Science and Technology*, 1(2):025006, 2020.

[17] Youngseog Chung, Ian Char, Han Guo, Jeff Schneider, and Willie Neiswanger. Uncertainty Toolbox: an open-source library for assessing, visualizing, and improving uncertainty quantification. *arXiv preprint arXiv:2109.10254*, 2021.

[18] Dan Levi, Liran Gispan, Niv Giladi, and Ethan Fetaya. Evaluating and calibrating uncertainty prediction in regression tasks. *Sensors*, 22(15):5540, 2022.

[19] Richard P. Brent. An algorithm with guaranteed convergence for finding a zero of a function. *The computer journal*, 14(4):422–425, 1971.

[20] Johannes Gasteiger, Muhammed Shuaibi, Anuroop Sriram, Stephan Günnemann, Zachary Ulissi, C Lawrence Zitnick, and Abhishek Das. GemNet-OC: developing graph neural networks for large and diverse molecular simulation datasets. *arXiv preprint arXiv:2204.02782*, 2022.
